# A Public Health Service-Learning Capstone: Ideal for Students, Academia and Community

**DOI:** 10.3389/fpubh.2019.00010

**Published:** 2019-01-29

**Authors:** Sara L. C. Mackenzie, Deborah M. Hinchey, Kathryn P. Cornforth

**Affiliations:** ^1^Department of Health Services, University of Washington, Seattle, WA, United States; ^2^Department of Biomedical and Health Sciences, University of Vermont, Burlington, VT, United States; ^3^Carlson Leadership and Public Service Center, University of Washington, Seattle, WA, United States

**Keywords:** high-impact practice, curriculum, service-learning, culminating experience, capstone, undergraduate public health education

## Abstract

Undergraduate public health degree programs strive to educate students to improve the health of communities. As such we have an obligation to develop curricula that push students to think critically about their perspectives, examine assumptions, and provide supported opportunities to apply their academic learning. In addition, curricula ideally develop and nurture students' sense of civic responsibility. Community-engaged learning provides opportunities for students to interact with populations with a range of needs and different perspectives. Students need to be prepared to engage ethically and respectfully, while thinking critically about and reflecting on their roles in these communities. Service-learning is a high-impact practice that combines community service with structured academic learning, including preparation, and reflection. In line with public health community-based work, a key aspect of service-learning is the intentional development of community partnerships to ensure that students are filling the needs defined by the communities themselves. Accreditation criteria may guide what is taught but say little about how it should be taught. However, how we teach matters. Service-learning is a high impact practice that not only aligns well with the goals and objectives of an accreditation required culminating senior experience but shares many of the values of the discipline of public health. This paper analyzes the use of service-learning in the development and delivery of the University of Washington School of Public Health undergraduate Public Health-Global Health majors' culminating experience. We describe the course learning objectives, structure, and assessment tools. In addition, we present quantitative and qualitative results on the impact of the course. We argue that it is feasible, sustainable, and beneficial to students and communities when the high impact practice of service-learning is used in delivery of a culminating senior experience.

## Introduction

Undergraduate public health degree programs strive to develop students who are competent to improve the health of communities. The Council on Education in Public Health (CEPH) developed requirements for accredited undergraduate baccalaureate programs (1). The domain areas and competencies guide *what* programs should teach students in order to ensure that they are adequately prepared to enter the workforce or to begin graduate education upon degree completion (1). However, the domains and competencies say little about *how* to structure curricula or deliver course content to achieve the intended results. Intentional consideration of how teaching methodologies are integrated into courses and across curricula provides an opportunity to provide purposeful student progression to achieve intended results (2–4).

High impact practices are approaches to content delivery that have been shown to increase educational outcomes at the college level (3, 4). While utilization of high impact practices benefits all students, the positive results in regards to GPA, probability of retention and completion are especially significant for students from communities historically underserved in higher education (3–6). High impact practices can be implemented at the course level—such as collaborative projects, service-learning, and study abroad. Other practices are at the curricular design level—such as learning communities, common intellectual experiences, and writing intensive courses.

The Bachelor of Arts and Bachelor of Science Public Health-Global Health majors at the University of Washington incorporate multiple high impact practices across the curriculum. After a twice-a-year application process, we welcome a diverse group of students who progress, in general, as a cohort through a 2-year, sequential, required series of courses. The purposeful pathway allows intentional development of competencies and allows scaffolded and repeated practice opportunities for students to progress to higher levels of challenge and engagement. For example, competence in public health writing is developed across the curriculum with all required courses building on prior course assignments and skills (7). In this paper, we present arguments for why the high impact practice of service-learning aligns with the intended learning outcomes and goals of an accreditation required culminating capstone experience.

## Background and Rationale

A senior culminating experience or capstone course requires seniors to apply what they have learned throughout their educational program. A culminating experience is, in and of itself, a high impact practice because it requires students to integrate and apply their learning (3, 4). Given the complexity of public health issues and the fact that public health work occurs in community, the service-learning model, when implemented well, is a practice designed to foster transformative student and community development (8). Students engage with community partners and are challenged to critically self-reflect, synthesize, and apply their public health learning, analyze ethical and civic situations, and, in partnership, work toward community action.

## Service-Learning Framework and Principles

“**Service-learning** should be about social change, not just filling a gap in services. It should be about questioning the conditions in society that create the need for service in the first place and seeking to alter those conditions” (8).

Service-learning is a pedagogy that aligns with public health discipline values. In fact, one can almost substitute the words “public health” for “service-learning” in this definition. Traditional service-learning emphasizes reciprocal and intentional work focused on community-identified needs. Incorporating components that require students to analyze and address power dynamics and adopt a social justice lens moves the service-learning pedagogy to what is referred to as “critical service-learning.” The reframing pushes students to do more—to analyze and challenge the power structures and systems of oppression that contribute to ongoing community needs. Students are encouraged to consider their role as agents of change (9–11).

Regardless of student background (prior lived and field experience), critical service-learning methods enhance cultural humility and students' abilities to work within and across communities (12). Service-learning balances academic and field experience creating a triad relationship between student, faculty and community agency. Teaching faculty frame the experience as a partnership and ask students to work with community partners to build on the strengths and knowledge present within the community. Intentional academic preparation requires students to draw and reflect on their prior academic learning and consider how it might apply in their field site. While the communities served and specific projects differ, academic exploration pushes students to critically reflect on and analyze the structures and power systems that create a need for service in the first place—an essential component of “critical” service-learning (9, 11, 13).

This pedagogy allows students to work in partnership with communities while simultaneously engaging in a structured academic and reflective classroom experience. Critical service-learning pedagogy develops the personal, civic, and academic capacities of public health students while directly benefiting the communities with whom they work (12, 14, 15). The integrative and applied learning aspects of critical service-learning make this an ideal high impact practice for achieving the culminating experience competencies.

## Capstone Competencies

The service-learning capstone is required for all majors and fulfills the CEPH culminating experience requirement necessary for program accreditation. In addition, the capstone learning objectives map to domain and competency areas identified as essential in the 2016 CEPH standards (1) for program accreditation. The learning objectives are high Bloom's level learning as students are expected to apply what they have progressively practiced through their required courses. Synthesis of learning is necessary to successfully work with agencies. By the end of the capstone, students are able to:

- Research and understand the role, structure and function of, and the population served by, a community-based agency
- Analyze the systemic causes and impacts of a public health problem on a population
- Map community resources, assess community resources, and synthesize community strengths and gaps
- Develop and communicate an action-oriented approach to address a community-identified public health problem
- Systematically apply prior public health knowledge to experiences within a service placement
- Evaluate personal attitudes and approaches to working with diverse communities and examine the impact of service on learning and communities
- Communicate public health content in oral and written form
- Effectively work in a diverse environment through interpersonal skill building, conflict resolution, and practical problem solving.

## Learning Environment

The University of Washington is on the quarter system and our capstone course spans two sequential quarters or 20 weeks. There are three offerings each year of the capstone (Fall/Winter, Winter/Spring and a Summer intensive). The pedagogical format is in person. The course was developed by a core faculty member in collaboration with the program director and is led now by a single instructor with in-classroom support of graduate teaching assistants. The course instructor works year round to develop and maintain relationships with community partners in coordination with the University of Washington Carlson Leadership & Public Service Center. The center provides support for community-based learning pedagogy and supports campus-community partnerships.

During the course, students meet weekly in class for a 110 min session with faculty. The course includes three key components:

1. **Academics:** The structured academic component ensures students are synthesizing and applying their academic learning. In addition, it creates a structured environment to support students in examining and reflecting on identity development and issues of power, privilege, and oppression. Assignments and in-class activities encourage student reflection on their implicit biases, backgrounds, and the lens through which they see the world. Table 1 illustrates the assignments and how they are structured across the 20 week curriculum. Weekly in-class sessions and scaffolded assignments develop students understanding of context (agency), population, and assets in and problems experienced by the population. Students develop an evidence base for the analysis of the systemic causes of a public health issue facing the populations with whom they are working. Their work culminates with a concrete and actionable project.
  Students produce an agency overview, including looking at the mission, vision, organizational chart, budget, and funding models for the agency. Students are regularly faced with situations on-site that require ethical decision-making. The pre-field work presents scenarios for students to work through. In both the service positions and the academic work, students are challenged to work independently and as part of a team to assess, analyze, and address pertinent health issues facing the populations with whom they are working. Professional development is embedded throughout the duration of the capstone course, and includes guest lectures by career specialists, resume workshopping, and professional communication. Academic assignments align with and assess student progress in achieving the identified course competencies. Assignments are graded using rubrics and ensure that, regardless of the site placement, students make connections to public health issues and broader course competencies. Students must demonstrate a thorough understanding of the material in order to pass the course.
2. **Community Service**: In the first 2 weeks of capstone, community partners present an overview of their work, populations served, community need, and field opportunity. Students select a placement that aligns with their personal goals and interests. A sampling of community agencies and projects are presented in Table 2. Students spend ~5 h per week over the course of 10 weeks at their field site for a minimum of 50 h of service. The first weeks of the capstone experience are focused on preparation and the last weeks reserved for culminating reflection and production of academic work related to the service experience. Students often continue working with their service site beyond the required number of weeks.
  Students are placed in small teams within the organization so that each site has at minimum three students. Students are not required to do their service at the same time as their peers but the team approach creates a group experience. Work within the organization is designed and supervised by the community partner organization. Students are supported to focus on community-articulated needs in their service hours. The service experience is framed as an opportunity to network with professionals working in the field and students have experiences in class and field which prepare them to network successfully. Site supervisors provide feedback to course instructor on student performance in particular as relates to professionalism and ethical behavior.
3. **Reflection:** Reflection is a critical component of service-learning and occurs throughout. Early reflection activities are designed to prepare students to enter the community. Our academic responsibility to our community is to ensure students enter communities aware of their own roles in power and privilege dynamics. During the field experience, reflection activities focus on supporting students in analyzing what they are observing and experiencing in the community. The closing reflective experiences as students to consider how the experiences has impacted them. The “what, so what, now what” experiential learning cycle is emphasized throughout (16, 17). Reflective activities and writings are not graded for content but are required components of the course.

**Table 1:** Academic activities and goals assessed.

**Assignment**	**Task and goal**
Population demographic profile and public health issue identification	One-page information sheet highlighting city, county and state-wide data and providing an overview of the population served by agency, including relevant public health issues
Agency/site overview	Oral presentation to class after an analysis of the structure, function, and funding of agency
Literature review	Written literature review providing an evidence base clearly demonstrating the causes and impacts of identified public health problem on population served by agency
Stakeholder interviews	Identification of and engagement with key stakeholders addressing public health problems and possible interventions in the community
Community asset mapping	Windshield survey of geographic area in which agency situated to include environmental factors that influence the lives of population served
Best practices and current intervention inventory	Evaluation of what is currently being done to address identified public health problem, including best practices and local, state and national efforts
Final project: policy, program or advocacy intervention	Development of a proposal recommending a population-level approach to address the public health problem. Recommendations can be either: (1) policy analysis (2) programmatic intervention or (3) advocacy plan

**Table 2:** A sampling of the range of service positions.

**Organization-type**	**Description of student role**
Public Health Department-Education and Outreach	Students conduct door-to-door outreach, facilitate CPR trainings, attend community events and develop marketing materials focused on improving the interface between vulnerable populations and 911.
Agencies Addressing Poverty Alleviation	Students work at a drop-in free tax completion program connecting individuals and families to entitlement benefits (food stamps, childcare assistance, WIC) based on income eligibility.
Hospital Affiliated Organizations	Students develop relationships with and provide companionship to patients in a long-term care facility; some students also do light housekeeping for house-bound residents in a public housing community.
Agencies Addressing Basic Needs—food, sanitation, shelter, and safety	Students build relationships with guests at programs for individuals experiencing homelessness, staff shelter and food bank programs and contribute to supportive shelter environments for survivors of domestic violence.
Agencies Addressing Inequities in Educational Outcomes	Students tutor English-language learners, first generation children, immigrants applying for citizenship, and children in afterschool programs in low income communities.

## Results and Impact

At this time, 899 students have completed the service-learning capstone. Students must complete all service hours, participate actively in-class (through attendance and group engagement), and demonstrate a thorough understanding of the material in order to pass the course. Students have provided upwards of 45,000 h of service to the community and produced multiple resources for partner organizations–from academic literature reviews of best practices to training videos for staff to community asset maps that are usable by clients of the organizations.

A 2018 student survey assessed student perception of the culminating experience. The online survey was administered to 150 students in class during the last week of the quarter. The response rate was 90 percent. Table 3 shows the questions and response rates from the survey. The majority of students felt that the capstone experience required them to apply prior learning, analyze systemic causes contributing to public health problems and apply this to formulate an approach to problem solving. In addition, to be successful the majority of students felt they had to evaluate their personal attitudes and approaches to working in communities and they were challenged to be professional and ethical in their behavior. The majority of students also felt they developed skills in teamwork, oral and written communication, accessing and interpreting literature, and systems thinking.

**Table 3:** Student perception of capstone learning and application.

	**Strongly agree**	**Agree**	**Neutral**	**Disagree**	**Strongly disagree**
**TO BE SUCCESSFUL IN CAPSTONE, IT WAS NECESSARY FOR ME TO:**					
Apply prior public health learning/knowledge to my experiences within a service placement	54 (40.0%)	59 (43.7%)	15 (11.1%)	3 (2.2%)	2.9 (3.0%)
Effectively analyze the systemic causes and impacts of a public health problem on a population	75 (55.6%)	52 (38.5%)	5 (3.7%)	1 (0.7%)	2 (1.5%)
Formulate an approach to comprehensively address a public health problem	71 (52.6%)	56 (41.5%)	5 (3.7%)	1 (0.7%)	2 (1.5%)
Evaluate personal attitudes and approaches to working in diverse communities	77 (57.0%)	48 (36.6%)	7 (5.2%)	1 (0.7%)	2 (1.5%)
Be professional and practice ethical behavior	90 (66.7%)	34 (25.2%)	7 (5.2%)	2 (1.5%)	2 (1.5%)
**THE CAPSTONE ALLOWED ME TO DEVELOP OR STRENGTHEN THE FOLLOWING SKILLS:**					
Working in groups	61 (45.2%)	57 (42.2%)	13 (9.6%)	3 (2.2%)	1 (0.7%)
Oral communication	55 (40.7%)	53 (39.3%)	21 (15.6%)	5 (3.7%)	1 (0.7%)
Written communication	69 (51.1%)	53 (39.3%)	11 (8.2%)	1 (0.7%)	1 (0.7%)
Accessing and interpreting academic literature	78 (57.8%)	47 (34.8%)	9 (6.7%)	0 (0)	1 (0.7%)
Effectively work in a diverse environment	78 (57.8%)	45 (33.3%)	8 (5.9%)	2 (1.5%)	2 (1.5%)
Systems/upstream thinking	74 (54.8%)	50 (37.0%)	10 (7.4%)	0 (0)	1 (0.7%)
Critical thinking and practical problem solving	73 (54.1%)	52 (38.5%)	8 (5.9%)	1 (0.7%)	1 (0.7%)

A key function of service-learning is the development of civic responsibility and community engagement in students. Table 4 shows results of a survey question which asks students to consider how the capstone impacted their sense of commitment and responsibility to communities. Twenty-five percent of students felt the capstone instilled this sense of commitment and responsibility, whereas sixty-six percent felt it strengthened an existing sense of commitment and responsibility. Only eight percent of students felt the experience did not change their sense of commitment and responsibility.

**Table 4:** Capstone impact on student commitment and responsibility to make a difference in communities.

**Which of the following best describes your experience. participation**	
**in capstone…**	
Instilled a sense of commitment/responsibility to make a difference in communities	34 (25.2%)
Strengthened my existing sense of commitment/responsibility to make a difference in communities	89 (65.9%)
Did not change my sense of commitment/responsibility to make a difference in communities	11 (8.2%)

We have used informal, regular meetings to assess the impact on community. The majority of our agencies have continued to request public health students once they partner with us for capstone. Many of our partner agencies have been with us since 2013. These ongoing relationships and partner feedback on student employability are important ways for us to assess student preparation for the workforce and program structure. Sample quotes from partners include:

- “Students have helped us in many ways. They have done data collection, human connection, helped us when we are short on staff and doing other activities, keeping the facility function… keeping the facility running…” site supervisor
- “Their willingness to place themselves in uncomfortable situations makes them a lot more ready to do things that other people may not be able to. They are very eager to learn about the challenges that the (homeless) women go through. They bring a certain sensitivity and skill that sometimes you can't teach…” volunteer coordinator.

The theme of public health students being able to extend the reach and hours of community agencies is common. For example, in an ongoing partnership since 2013, students at one capstone site have assisted emergency responders in educating 12,000 limited English proficient residents on 9-1-1 and CPR through workshops and knocked on over 2,000 doors in door-to-door outreach. Another site requested student engagement during the summer in order to meet their agency needs. In response, we began offering a summer intensive capstone so the organization could provide summer hours for clients.

## Discussion

The service-learning model is a sustainable, high impact practice that aligns well with the instructional needs of a culminating senior capstone course and the values of the public health discipline. The practical implications of our model is that it is flexible enough to serve thirty students at program inception 6 years ago and expandable to now meet the needs of over 500 students. Our results show that the practice is effective at engaging students in high level work. In addition, for the majority of students, the experience instilled or strengthened their sense of commitment and responsibility to the community.

While students appreciate experiential opportunities and service-learning is well-received, we have had to balance the time, flexibility, and effort components of academic and community work due to demands on and complexity of student lives. For example, for several years a service site involved students teaching in local high schools about public health. However, the rigidity of high school class times and shifting schedules during quarter changes created ongoing challenges for our students that could not be overcome and we had to let go of this service site. We have found being realistic and transparent about travel time, schedule flexibility, and student obligation at sites is critical for ongoing mutual benefits.

A challenge that sometimes arises is a student requesting to develop their own service site instead of working with an agency with whom we have a relationship. The motivation behind these requests are almost always student driven with different goals than intended for capstone. We have chosen to not allow this for several reasons. A critical component of capstone is the balancing of student needs with community needs. In setting up their own sites, students tend to focus on their own needs. Additionally, the professional and applied skills incorporated into the team-based course structure are essential to preparing students for post-graduation success. We do encourage these students to set up a credit-bearing internship with alternative sites as an internship is typically more focused on student driven objectives. If they choose this option, it is in addition to capstone.

Another powerful aspect of our model is that we utilize a team-based approach to the assignment of students to sites. Each site has at minimum three students working at it. The initial goal of the structure was to support students building skills working on site projects together and conflict resolution. However, further benefits to the team/group model quickly became clear. The shared student experience emulated a real-world work experience, and students were able to create a community of practice. Students appreciated having peers with whom they could discuss experiences, examine their reactions, and support each other to push their comfort zones. Additionally, the team-based approach helps with site coordination as we need less sites to accommodate our large number of students.

The identification, development and maintenance of community partnerships is critical to the success of service-learning. Because of this, we invest in designated faculty time for relationship building. We are fortunate that community agencies will often approach the UW Carlson Center or our faculty when they identify a need for students. As in many communities, we must be sensitive to not overburdening community partners as undergraduate and graduate students from many U.W. schools and colleges, in addition to other programs in the area, regularly perform community work through service-learning, internships, and pratica.

We tend to select service-learning agencies addressing a broad range of social determinants of health instead of governmental public health agencies. Additionally, it is imperative to provide students with a choice of sites that serve varied populations and offer diverse experiences. The maintenance of sites requires significant facetime between instructor and community partner (14). We have found this to be true and have regular meetings between faculty, agencies, and the Carlson Center staff to develop shared expectations around academic and field work. Agency partner engagement in planning supports the entire experience. When sites are aware of the academic components, not only can they help to guide students in topic areas but they benefit from the work. For example, student literature reviews can be guided and used by agencies. On many occasions, agencies will use student developed work in the professional development of agency staff.

Another challenge arose with the rapid growth of our majors and consideration of a culminating service experience. Our school also graduates approximately one hundred and fifty Masters in Public Health students a year. The majority seek local placements for their required 120-h practicum experience. Unlike undergraduates, MPH students are expected to contribute substantial knowledge and skills while working with agencies and require significant on-site mentorship. When constructing experiences for undergraduates, we needed to ensure that we were not increasing the already great supervisory burden on partner agencies. Additionally, it was essential to work with partners clarify how their needs aligned with the skill sets of different learners to create best fit scenarios. We find that the needs of non-governmental social service organizations that address public health issues are often more in line with the skill sets of undergraduates and offer robust opportunities to engage students in big public health questions.

## Conclusion

Service-Learning, and in particular “critical” service-learning is a high impact practice that serves well as a method of delivery for the culminating senior experience in public health major degree programs. The approach requires students to synthesize and apply their learning in the community. In addition, students analyze and challenge the power structures and systems of oppression that contribute to ongoing community needs. Students consider their role as agents of change. The intentional engagement with community agencies creates a sustainable and action-oriented model for education.

## Author Contributions

All authors contributed to the conception of the course. DH developed the course content. SM and DH co-wrote early drafts. All authors provided content expertise, contributed to manuscript revision, read, and approved the submitted version.

### Conflict of Interest Statement

The authors declare that the research was conducted in the absence of any commercial or financial relationships that could be construed as a potential conflict of interest.
